# Radionuclide-based molecular imaging allows CAR-T cellular visualization and therapeutic monitoring

**DOI:** 10.7150/thno.56989

**Published:** 2021-05-03

**Authors:** Fuqiang Shao, Yu Long, Hao Ji, Dawei Jiang, Ping Lei, Xiaoli Lan

**Affiliations:** 1 Department of Nuclear Medicine, Union Hospital, Tongji Medical College, Huazhong University of Science and Technology, Wuhan 430022, China.; 2 Hubei Key Laboratory of Molecular Imaging, Wuhan 430022, China.; 3 Department of nuclear medicine, Zigong First People's Hospital, Zigong 643000, China.; 4 Nuclear Medicine and Molecular Imaging Key Laboratory of Sichuan Province, Luzhou 646000, China.; 5 Department of Immunology, School of Basic Medicine, Tongji Medical College, Huazhong University of Science and Technology, Wuhan 430030, China.

**Keywords:** chimeric antigen receptor T cell, molecular imaging, side effects, therapeutic monitoring, direct labeling, reporter gene, endogenous cell

## Abstract

Chimeric antigen receptor T cell (CAR-T) therapy is a new and effective form of adoptive cell therapy that is rapidly entering the mainstream for the treatment of CD19-positive hematological cancers because of its impressive effect and durable responses. Huge challenges remain in achieving similar success in patients with solid tumors. The current methods of monitoring CAR-T, including morphological imaging (CT and MRI), blood tests, and biopsy, have limitations to assess whether CAR-T cells are homing to tumor sites and infiltrating into tumor bed, or to assess the survival, proliferation, and persistence of CAR-T cells in solid tumors associated with an immunosuppressive microenvironment. Radionuclide-based molecular imaging affords improved CAR-T cellular visualization and therapeutic monitoring through either a direct cellular radiolabeling approach or a reporter gene imaging strategy, and endogenous cell imaging is beneficial to reflect functional information and immune status of T cells. Focusing on the dynamic monitoring and precise assessment of CAR-T therapy, this review summarizes the current applications of radionuclide-based noninvasive imaging in CAR-T cells visualization and monitoring and presents current challenges and strategic choices.

## Introduction

Immunotherapy is becoming the mainstay of cancer treatment after conventional approaches (surgery, radiotherapy, and chemotherapy) have failed. Immunotherapeutic strategies include vaccines, immune checkpoint blockade, antibody-drug conjugates, radionuclide-labeled antibodies, and, most recently, adoptive cell therapies (ACT) 1. The development of ACT has mainly focused on the construction of a chimeric antigen receptor (CAR)-T cell 2, 3. CAR-T cells' antitumor activity is the result of the formation of an immune synapse with target cells, leading to expression of pro-apoptotic ligands and accompanied by the release of cytotoxic perforin and granzyme and the secretion of pro-inflammatory cytokines (such as interferon [IFN]-α, IFN-γ, and interleukin 2 [IL-2]) by the T cells. This results in the activation of endogenous immune responses 3. CD19-targeting T cells, the most representative form of CAR-T therapy, uses autologous T cells engineered to express the CD19 antigen receptor. They attack CD19-positive malignancies with high affinity, and produce durable response 4, 5, resulting in better curative effect in patients with leukemia or lymphoma 6-8.

However, several clinical trials have shown that CAR-T therapy in solid tumors remains unsatisfactory. The main reasons may be related to the difficulties of defining tumor specific targets, the limited CAR-T cells trafficking to the tumor site, and the immunosuppressive microenvironment of solid tumors 2, 9-13. Several strategies are being dedicated to serve these problems 2, 13-17. Providing that these obstacles can be overcome, CAR-T therapy may have great potential to treat solid tumors.

Because CAR-T cells are a “living drug” and their migration, localization, infiltration, expansion, and persistence of CAR-T cells after infusion are dynamic processes 17. The safety of CAR-T cell therapy has always been a concern, as it is frequently accompanied by severe adverse effects, such as on-target off-tumor toxicity, neurotoxicity, and cytokine release syndrome. These are still the chief impediments to CAR-T therapy 18, 19. Dynamic monitoring of CAR-T cells would improve the understanding of cellular *in vivo* behaviour, which may allow optimization of the infusion timing and dose 20. It may also help avoid potential lethal systemic toxicity. Therefore, many questions pose great challenges for CAR-T monitoring. For example, can the transferred cells home to the tumor site? Can the cells infiltrate the tumor bed? How many cells infiltrate into the tumor? How long can the cells retention in the tumor? When did the expansion and contraction happen? Are the cells off-target to settle in extratumor tissues? Can the cells infiltrated in the tumor site play a tumor killing effect?

Current monitoring in CAR-T cell studies has mainly focused on disease response assessment, on CAR-T cell persistence, expansion, and effector function, and on serum cytokine (such as IFN-α, IFN-γ and IL-2) and immune marker levels 17. In solid tumors, the disease response assessment usually depends on the tumor size and morphological change detection by computed tomography (CT) and/or magnetic resonance imaging (MRI), which can provide information about the tumor burden, but the spatial information of infused T cells is undetectable 21, 22. Similarly, although peripheral blood tests can assay adoptively transferred T cells and associated cytokines or immune markers in the circulation, they are unable to show the spatial distribution and tumor-specific expansion of infused T cells, and the blood values measured may not accurately quantify the extent of tumor infiltration 17.

Surveillance of adoptively transferred CAR-T cells can also be achieved through tumor tissue biopsy, but the information obtained strongly depends on sampling, which often only involves a small part of the tumor tissue and cannot fully reflect T cells infiltration in entire tumor 21. The development of visualization tools that go beyond anatomical imaging and provide spatial information of cells, is key to achieving dynamic, non-invasive monitoring of therapeutic cells.

Molecular imaging is a novel and rapidly developing discipline combining molecular biology and medical imaging techniques to allow the visualization of biological processes 23-25. Different molecular imaging modalities have their own advantages and disadvantages. Optical imaging (OI), such as bioluminescence imaging (BLI) and fluorescence imaging (FLI), can offer great sensitivity at a lower cost and higher throughput 26. OI is suitable for* in vitro* studies and frequent small animal imaging 27, but several shortcomings block the clinical translation, such as lack of tomographic information, inability to image deep tissue, poor spatial resolution, and mass quantity (g to mg) of probe needed 26, 28, 29. Although MRI has a high spatial resolution and excellent soft tissue contrast, without ionizing radiation 30, 31, it is handicapped by the inherently relatively lower sensitivity in comparison to OI and nuclear imaging 32. Radionuclide-based imaging, including positron-emission tomography (PET) and single-photon emission computed tomography (SPECT) paired with CT (PET/CT, SPECT/CT) or MRI (PET/MRI) afford high sensitivity, multiple available probes, and a combination of quantitative physiological and tomographic information 33, 34. Despite the shortcomings such as radiation exposure, expense, and low spatial resolution, PET/SPECT imaging techniques remain the greatest clinical translation potential compared with OI and MRI 35-37.

**Figure 1:**
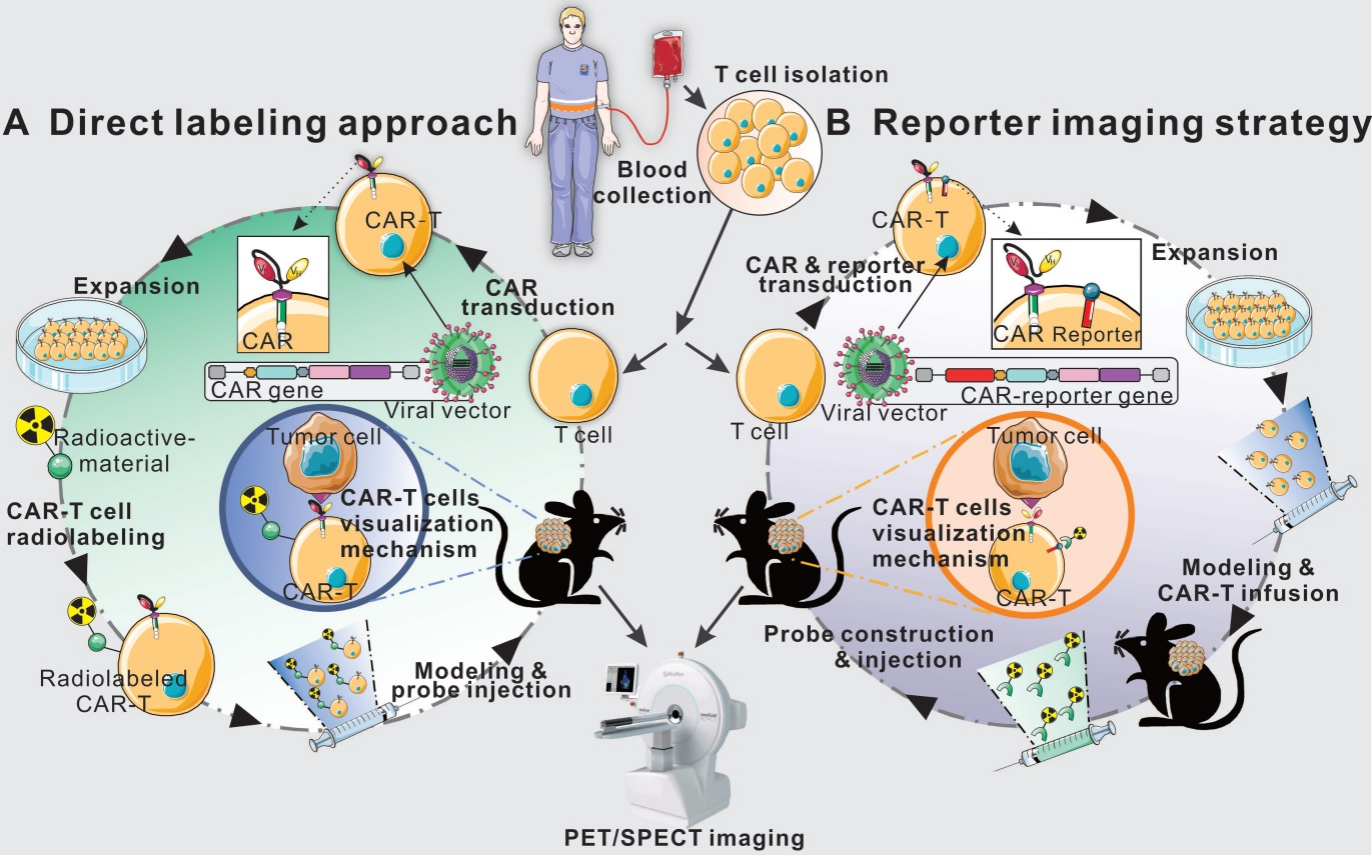
** Schematic diagram of direct labeling approach and reporter gene imaging strategy.** Blood is collected from patients or healthy donors, and peripheral blood mononuclear cells are isolated. Then, T cells are separated and harvested for further direct labeling or genetic modification. In the direct labeling approach (**A**), T cells are first transduced with a CAR-encoding gene to generate CAR-T cells. After expansion, CAR-T cells are radiolabeled with radioactive material, and then are infused into tumor-bearing mice. The radiolabeled CAR-T cells can be monitored by longitudinal PET/SPECT imaging. In the reporter imaging strategy (**B**), a recombinant gene for co-expressing CAR and reporter is transduced into T cells to generate reporter-CAR-T cells. After expansion, the reporter CAR-T cells are infused to tumor-bearing mice for treatment and longitudinal PET/SPECT monitoring.

So far, radionuclide-based T cells imaging techniques include two main categories, direct labeling approach, or the reporter gene strategy 35, 38. The former refers to the T cells are passively labeled with radionuclides or radioactive materials *in vitro* 39, 40, and the latter depends on the expression of an enzyme, receptor, or transporter by reporter gene-transduced T cells 41-45. Several studies in animal models, as well as clinical trials, have demonstrated that radionuclide-based imaging allow directly (*ex vivo* labeling) or indirectly (reporter gene) visualization of CAR-T cells *in vivo*, which would provide crucial information about the proportion of viable cells and their biodistribution (**Figure 1**). In addition, endogenous T cell imaging can reflect the cellular activation and functional status by targeting cell surface markers or key materials in the metabolic pathways 35, 46, 47, which could be applied as a supplementary strategy for the two main imaging techniques.

This review aims to provide an overview of the most recent developments in PET/SPECT imaging based on direct labeling and reporter gene strategy for the visualizing and monitoring of CAR-T cells, as well as the current applications of endogenous T cell imaging in the cellular field. The advantages and disadvantages of these imaging strategies were discussed and the key aspects of imaging strategy choice are summarized for looking forward the future progress.

## Direct labeling approach

As shown in **Figure 1**, T-cells derived from patients or healthy donors are transduced with a CAR-encoding gene to generate CAR-T cells. After expansion and characterization, the CAR-T cells could be *in vitro* labeled with radionuclide for imaging. The major advantage of direct labeling approach is the simple nature of the labeling process, which requires minimal manipulation of the cells. Recent publications 48-50 have shown that CAR-T cells can be radiolabeled directly using a range of materials such as small molecules and nanoparticles.

### Small molecule-based labeling

Two CAR-T cells, targeting transmembrane glycoprotein Mucin 1 (MUC1) and the extended ErbB network respectively, were developed by Parente-Pereira et al. CAR-T cells were passively labeled with ^111^In-tropolonate to allow high-resolution real-time cellular tracking by SPECT/CT imaging 48. When they were infused intravenously into tumor-bearing severe combined immunodeficiency (SCID) Beige mice (MUC1-targeting CAR-T for MDA-MB-435 breast cancer, and ErbB-targeting CAR-T for HN3 human head and neck squamous cell carcinoma), the engineered CAR-T cells distributed in the murine lungs, liver, and spleen without significant penetration into the tumor. When infused via either the intraperitoneal or subcutaneous route, the CAR-T cells remained mostly at the site of injection. This provided first-hand experience of direct cellular radiolabeling in CAR-T therapy and enabled successful tracking of cell migration *in vivo*.

**Figure 2:**
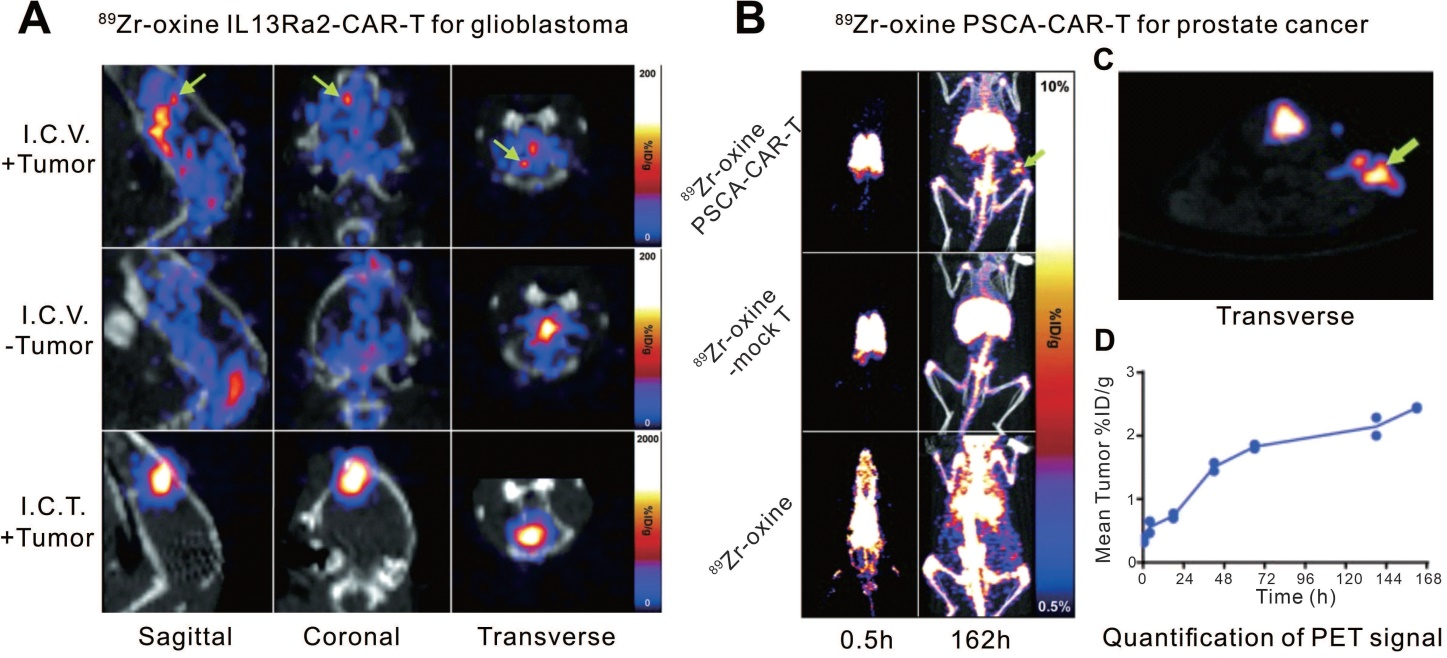
** CAR-T cellular visualization based ^89^Zr-oxine labeling. A.** Mice with (+Tumor) or without (-Tumor) glioblastoma in the left cortex were administrated ^89^Zr-oxine-labeled IL13Rα2-CAR-T cells injected into the cerebrospinal fluid via the right ventricle (I.C.V.) or directly into the tumor (I.C.T.). PET/CT images demonstrated activity accumulation in the tumor sites by I.C.V. (top row, green arrows) and I.C.T. (bottom row), whereas the activity is diffusely distributed in the ventricular system in tumor-free mice (middle row). **B.** Prostate cancer xenograft-bearing mice were treated with ^89^Zr-oxine labeled PSCA-CAR T cells (top row), ^89^Zr-oxine labeled untransduced T cells (middle row), and cell-free^ 89^Zr-oxine (bottom row), respectively. Pulmonary activity accumulation was observed in the mice treated with T cells, while the activity of the cell-free ^89^Zr-oxine was rapidly distributed throughout the blood at the early time point. Activity in subcutaneous xenograft (green arrow) was detected in mice treated with CAR T cells but not with mock T cells or ^89^Zr-oxine. **C.** The transverse image of subgraph B. **D.** Time-activity curve of the region of interest of xenografts in mice treated with ^89^Zr-oxine PSCA-CAR T cells. Adapted with permission from 52, copyright 2018 Society of Nuclear Medicine and Molecular Imaging.

Another small molecule, oxine, has been often used in the labeling and tracing of murine lymphocytes 51-53. Weist et al. 49 first used oxine for the radiolabeling of human-derived CAR-T cells. They engineered two different CAR-T cell lines (interleukin-13 receptor α2 [IL13Rα2]-CAR-T and prostate stem cell antigen [PSCA]-CAR-T) and labeled them with ^89^Zr-oxine. ^89^Zr-oxine-CAR-T cells maintained *in vivo* cytokine production, migration, tumor cytotoxicity, and* in vitro* anti-tumor activity at a labeling density of 70 kBq per million cells. The radiolabeled IL13Rα2-CAR-T and PSCA-CAR-T cells were administered to glioblastoma- and prostate cancer-bearing nonobese diabetic severe combined immunodeficient (NSG) mice, respectively, and they were visualized by PET/CT imaging with relatively high specificity (**Figure 2**). Additionally, since ^89^Zr has a long physical half-life (78.4 h), the infused CAR-T cells could be traced and monitored dynamically on PET for up to 6 d. We cannot directly compare the two abovementioned studies because the nuclides, linkers, animal models, and modalities are different. However, Zr-89 is suitable for PET, which outperforms SPECT due to higher resolution and quantitative nature. Furthermore, compared to In-111, Zr-89 labeled cells can implement relatively longer imaging time window (96 h versus 6 d). Therefore, we believe the imaging performance of Zr-89 labeled CAR-T seems to be slightly better.

### Nanomaterial-based labeling

Gold nanoparticles (GNPs) have been found appropriate for intracellular retention 54. This property was utilized by Bhatnagar and colleagues 50 for CAR-T cell radiolabeling and tracking. GNP were labeled with ^64^Cu^2+^ using the macrocyclic chelator (1,4,7,10-tetraazacyclododecane-1,4,7,10-tetraacetic acid, DOTA) and polyethyleneglycol (PEG) 2000 to construct GNP-^64^Cu/PEG2000, which was then electroporated into genetically modified CD19-targeting CAR-T cells by the *Sleeping Beauty* transposon/transposase system. The GNP-^64^Cu/PEG2000-loaded CAR-T cells were infused intravenously into healthy mice for PET imaging, demonstrating intense activity accumulation in the lungs at 10 min post-infusion. Subsequently, activity in the liver and spleen gradually increased. Although this study exhibited the feasibility of a nanomaterial-based strategy for *in vitro* labeling and* in vivo* imaging of CAR-T cells, it was limited to design an imaging strategy for tumor-bearing mice with T cell infusion and to provide information on whether T cells home to the tumor.

The abovementioned investigations confirm the feasibility of a direct labeling strategy for CAR-T migration detection in solid tumors. However, some inherent shortcomings hamper the clinical translation, including imaging signal dilution due to cell division and death, and signal aliasing due to dead labeled cells being consumed by phagocytes. Moreover, because a direct labeling strategy allows CAR-T cells to be radiolabeled only once *in vitro* before administration, radionuclides with relatively long half-lives, such as ^64^Cu (12.7h), ^111^In (67 h), and ^89^Zr (78.4 h), would be preferred to extend the imaging period. Because a treatment cycle of CAR-T therapy usually lasts for several weeks 55, 56, even if the imaging timeframe of the direct labeling approach can reach 96 h 48 or 6 d 52 after probe infusion, it is still insufficient for longitudinal imaging and obtaining information about the *in vivo* kinetics of CAR-T cells in later parts of the treatment cycle. In addition, nanomaterials such as GNPs may need to be introduced into cells as carriers of the radionuclide. Whether or not they may cause potential biological toxicity to CAR-T cells is a question that requires careful consideration.

## Reporter gene strategy

Compared to direct labeling, reporter gene imaging exhibits many advantages. First, genetic engineering and consequent stable genomic integration allow the reporter protein to be stably expressed in living transduced cells. Since dead cells no longer express the reporter gene product, the reporter probe will not bind to them, ensuring that all detected signals are from living cells 57. Second, cell division does not cause signal loss or dilution because progeny CAR-T cells also express reporter protein. Third, the transduction of reporter genes does not affect the viability, proliferation, or tumor killing ability of T cells *in vitro* or *in vivo* 58, 59. Fourth, the application of appropriate reporter/probe combinations can achieve repeated serial imaging without a restricted timeframe. Finally, based on the advantages of sequential imaging, radionuclides with shorter half-lives, such as ^68^Ga (67 min) and ^18^F (109.7 min) may be used for probe construction, therefore restricting ionizing radiation exposure to T cells and reducing potential radiation toxicity.

In light of these advantages, the reporter gene strategy has been used for ACT mapping and monitoring 41, 60-63. Recently, researchers have applied this strategy to CAR-T. Because the engineering of CAR-T cells requires DNA transduction *in vitro*, some researchers construct a recombinant DNA to co-express a CAR for tumor-specific targeting, and a reporter for corresponding PET/SPECT probe binding and cellular imaging in animal models or clinical applications.

### Enzyme-based reporter gene

The only reporter gene currently used for CAR-T imaging in human patients is an enzyme-based reporter gene, herpes simplex virus 1 thymidine kinase (HSV1-tk), which enables PET imaging with several radiolabeled probes such as ^124^I/^125^I/^131^I/^14^C-labeled 2-fluoro-2-deoxy-1-β-D-arabinofuranosyl-5-iodouracil (FIAU), ^18^F labeled 9-(4-fluoro-3-hydroxymethylbutyl)guanine (FHBG) 64, 65, 2′-deoxy-2′-fluoro-5- ethyl-1-β-D-arabinofuranosyl-uracil (FEAU) 66 and 9-(3-fluoro-1-hydroxy-2-propoxymethyl)guanine (FHPG) 65, 67, 68. HSV1-tk-based reporter gene imaging has been used to monitor the biodistribution and homing of various transduced cells, such as administrated cytotoxic T lymphocytes (CTLs) in tumor xenografts 69 and inflammation models 70, grafted neurons in brain injury 71, and transplanted stem cells in ischemic heart disease 72. Dotti et al. 73 reported that infused sr39 mutant HSV-1 tk gene-transduced T lymphocytes could be visualized by ^18^F-FEAU PET imaging in nonhuman primate models, which supports their potential application in humans. Ponomarev et al. have employed HSV1-tk reporter-based imaging to monitor the T-cell receptor (TCR)-dependent nuclear factor of activated T cells (NFAT)-mediated activation of T cells, which suggests that the monitoring of CAR-T cell activation can be achieved through HSV1-tk reporter-based imaging 74.

**Figure 3:**
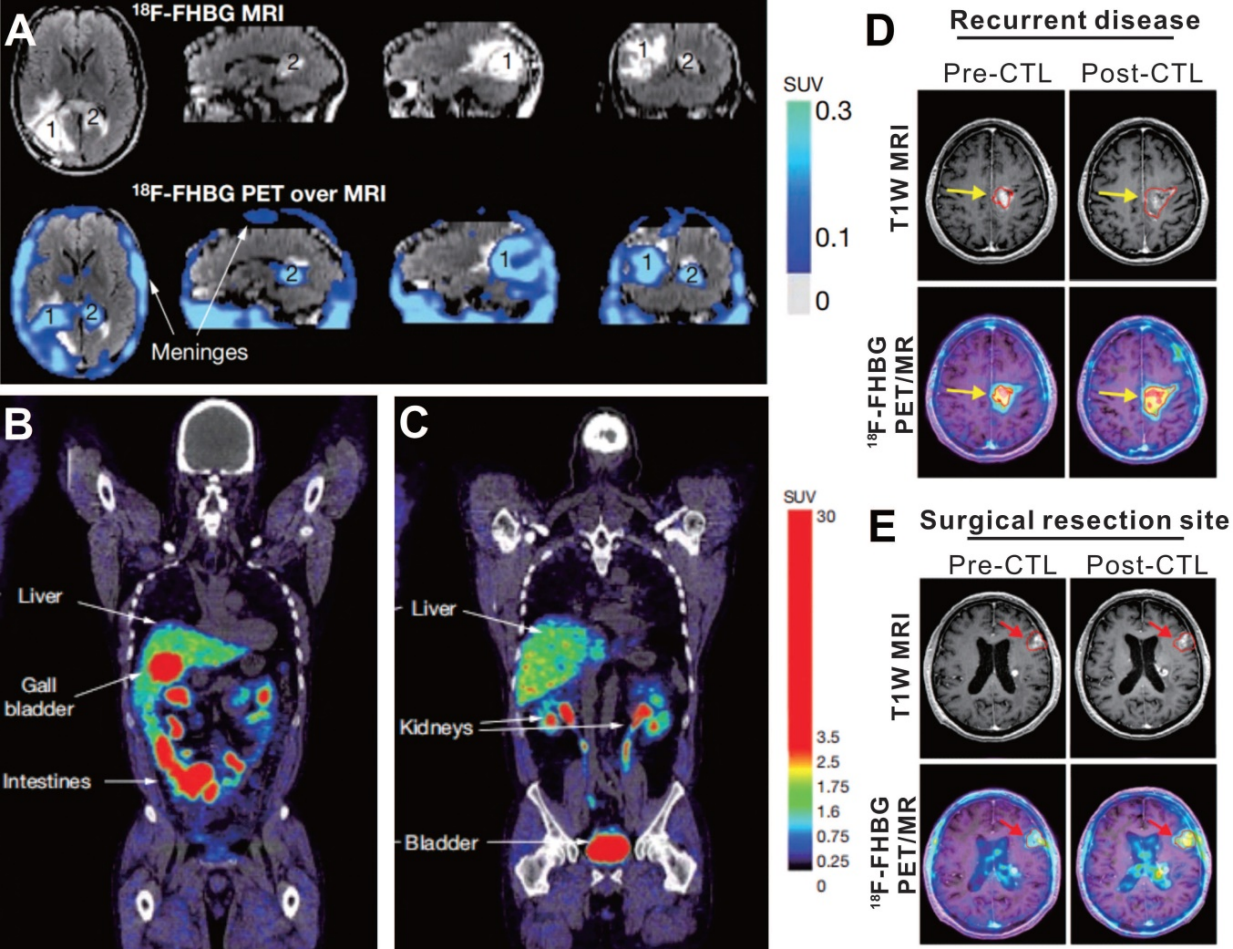
**^18^F-FHBG PET imaging based on HSV-1 tk reporter enables CAR-T cell visualization in patients with glioblastoma. A.** Images of brain MRI and ^18^F-FHBG PET/MRI superposition in a patient who received IL-13- and HSV-1 tk- expressing T cells. Both the surgically resected tumor site (lesion 1, which received CAR-T injection) and the unresected tumor (lesion 2, which did not receive CAR-T injection) had higher activity in contrast to the normal brain background. **B-C.** Sites of physiologic uptake of ^18^F-FHBG PET/CT included the liver, gallbladder, intestines, kidneys, and bladder. **D-E.** Pre- and Post-CTLs ^18^F-FHBG PET/MR images in recurrent disease (**D**, received CAR-T injection) and at untreated tumor (**E**) sites in a 60-year-old man with multifocal left hemispheric glioma. ^18^F-FHBG PET images showed the recurrent tumor and untreated tumor (B2, red arrows) had obvious activity that increased after CTLs treatment. A-C adapted with permission from 64, copyright 2008 Springer Nature, and D-E from 77, copyright 2017 American Association for the Advancement of Science.

The first case using HSV-1 tk reporter gene imaging to visualize administered CAR-T cells was of a 57-year-old man with grade IV glioblastoma multiforme (NCT00730613), reported by Yaghoubi et al. 64 After gross tumor resection, a cumulative dose of 1 × 10^9^ therapeutic T cells (expressing HSV1-tk as the reporter gene, and interleukin 13 [IL-13] zetakine targeting for IL-13Ra2-expressing tumor) into the tumor site. The ^18^F-FHBG PET/MR images demonstrated activity trapped both in the surgically resected tumor bed and in unresected tumor (**Figure 3, A-C**), which was thought to correspond to T cells accumulation. This case suggests that ^18^F-FHBG can accumulate within glioma tumors 75, and should be able to detect transferred cells that express HSV1-tk. However, because of the lack of a critical baseline PET image before CAR-T cells injection, the authors could not confirm whether the ^18^F-FHBG activity in the tumor region originated from the CAR-T cells *per se* or from tumor-associated nonspecific uptake 76.

Based on these findings, Yaghoubi's group conducted a clinical trial (NCT00730613 & NCT01082926) with the same reporter imaging approach, which involved seven cases of recurrent high-grade glioma that were resistant to conventional therapies 77. The results showed that the total lesion ^18^F-FHBG activity (SUV_mean_ × volume of interest) in sites of tumor recurrence significantly increased on the post-CTLs PET scan compared with the pre-CTLs PET scan, which suggested CAR-T cells migrating to the tumor sites (**Figure 3D-E**). The work from Yaghoubi et al. lays the foundation for a reporter gene imaging strategy to monitor CAR-T therapy of solid tumors in clinical practice. One important limitation of the presented system is the uptake of ^18^F-FHBG in untreated tumors and tumor resection sites. Yaghoubi et al. explained that the retention of ^18^F-FHBG in tumors before CTLs infusion may result from slow washout of the radiotracer from the resection cavity or be a consequence of off-target retention in the tumor cells 77. This accumulation of radiotracer in the untreated tumors or the tumor resection sites pre-CTLs infusion does cause potential background interference, which makes it difficult to quantify the truly signal emitted from transduced T cells after CTLs infusion. Although the reporter gene imaging with HSV1-tk and its mutant has been introduced into the clinical trials, the potential immunogenicity poses a major risk. Using mammalian species reporter gene constructs could effectively reduce this risk 78, 79.

In addition to clinical trials, a few recent studies have used reporter gene strategy for PET/SPECT imaging to verify the feasibility of tracking CAR-T cells in animal models (**Table 1**). Sellmyer's group developed a high-sensitivity PET reporter/probe pair, *E. coli* dihydrofolate reductase (eDHFR) and ^11^C/^18^F-fluoropropyl-trimethoprim (TMP) 80, 81. TMP, the probe precursor of this reporter system, is an inexpensive and widely available anti-biotice with and established toxicity profile. The absorption, distribution, metabolism, and excretion of TMP are favorable with a relatively short blood half-life in humans, and it has a low serum protein binding ratio (approximately 50%) 82, 83. The previous work on ^11^C-TMP 80 showed this reporter system to have high sensitivity for visualizing transduced cells, which can detect as few as 3 × 10^5^ cells. Sellmyer et al. applied reporter imaging strategy to monitor CAR-T cells targeted to the GD2 disialoganglioside in murine osteosarcoma models 84. ^18^F-TMP PET/CT showed promising accumulation in eDHFR^+^ human colon carcinoma (HCT116) cell xenografts with physiologic uptake in the liver, kidneys, and intestines, whereas lower uptake was noted in the control eDHFR^-^ tumor, blood pool, heart, lungs, muscles, spleen, skin, and the brain 84. The GD2^+^ tumor-harboring mice were administered with eDHFR-expressing anti-GD2 CAR-T cells, and ^18^F-TMP PET/CT was performed to track the cells. The results illustrated the CAR-T cells mainly accumulate in the spleen at the early time point, and migrate to the tumor at the late time point. These findings provide temporal and spatial information for homing and infiltration of T cells. Notably, eDHFR is a smaller protein compared with HSV-tk (18 kDa *versus* 46 kDa) with fewer immunologically active epitopes measured by sequencing. Future studies might focus on modifications of the eDHFR/TMP complex to enhance the binding affinity and humanize or truncate the enzyme to minimize its potential immunogenicity, which could offer important advantages in terms of deepening the understanding of CAR-T kinetics and promoting clinical translation 85, 86.

### Symporter-based reporter gene

**Figure 5:**
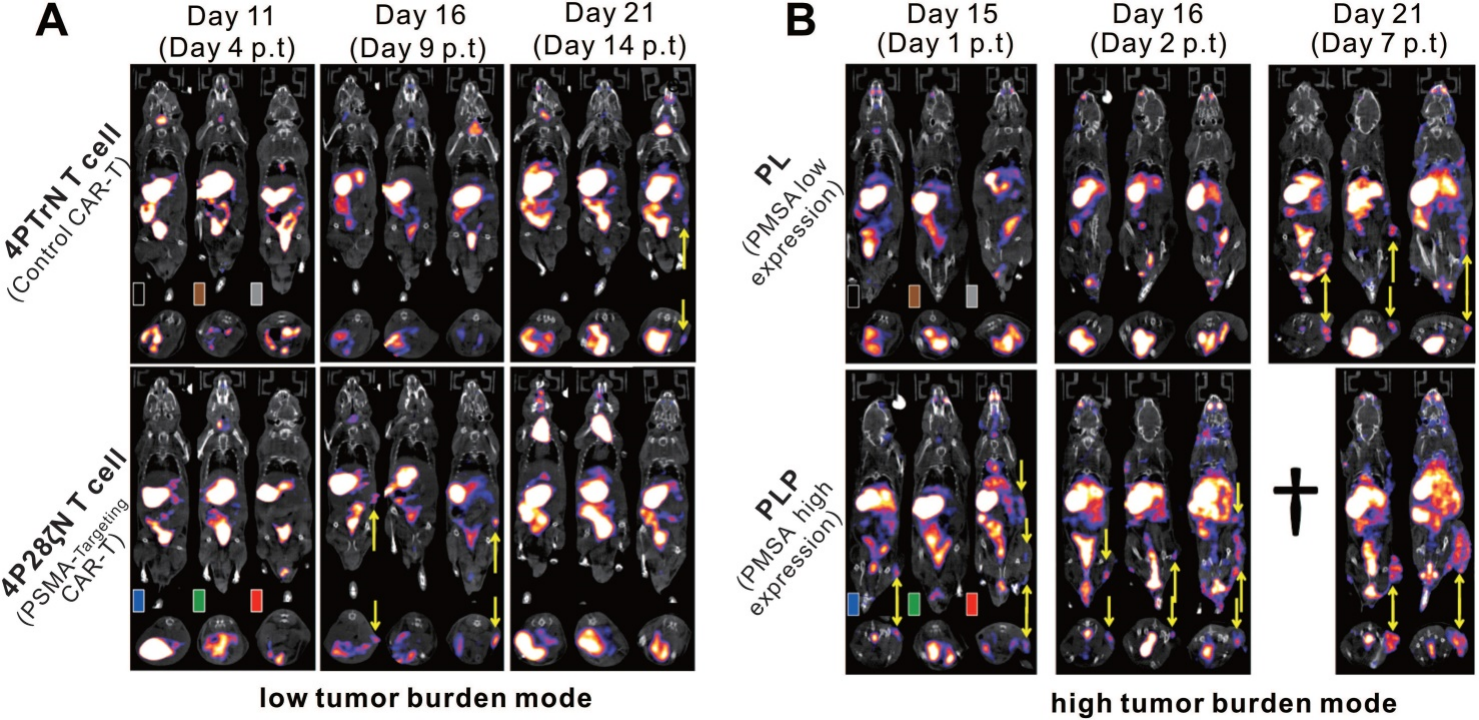
**CAR-T cellular visualization based on hNIS reporter SPECT/CT imaging. A**. ^99m^TcO_4_^-^ SPECT images of low tumor burden mice (imaged 7 d post tumor inoculation). Mice with PC-LN3-PSMA (PLP) cell subcutaneous xenograft were treated with control CAR-T (4PTrN^+^, upper row) cells and PSMA-targeting CAR-T (4P28ζN^+^, lower row) cells. No visible activity was detected in the tumors of all of the three control mice on the day 4 p.t and day 9 p.t, and only one had subtle activity in the tumor on the day 14 p.t. Although there is no detailed description in the original reference, the accumulation in the tumor (yellow arrows) of rightmost mouse on day 14 p.t might be due to the non-specific infiltration of T cells into tumor tissue 104. In comparison, significant SPECT signal was detected in the tumor of the mice treated with 4P28ζN^+^ CAR-T cells on day 9 p.t, and then the signal decreased on day 14 p.t as the tumor regression.** B**. ^99m^TcO_4_^-^ SPECT images of high tumor burden mice (imaged 14 d post tumor inoculation). Mice with PC-LN3(PL) cell xenograft (upper row) and PC-LN3-PSMA (PLP) cell xenograft (lower row) received 4P28ζN^+^ CAR-T cell infusion. Only subtle activity was detected on day 7 p.t in the PL tumors, indicating only a few CAR-T cells infiltration. In contrast, obvious SPECT signal was noted in the PLP tumors as early as day 1 p.t, which enhanced constantly till day **7** p.t (†=dead mouse). Adapted with permission from 58, copyright 2018 Springer Nature.

Human sodium-iodine symporter (hNIS) belongs to the sodium-dependent transporter family, which regulates the transmembrane transport of iodide and several other anions 87, 88. The hNIS is endogenously expressed in the thyroid and some other extra-thyroidal tissues including the gastric mucosa, salivary glands, and lactating mammary glands 89-91. In addition, hNIS is non-immunogenic, and is not internalized upon substrate uptake 92. Moreover, hNIS reporter imaging is suitable for PET and SPECT because NIS-expressing cells can effectively and specifically accumulate various radionuclides including ^99m^Tc, ^123^I, ^124^I, and ^131^I 90, 93. As an imaging reporter gene, hNIS has been well-used in tracking or assessing immune cells 94-96, regenerative cells 97-99, tumor cells 100-102, and cellular therapies 58, 103 in animal studies and clinical trials. Based on these attributes, the hNIS is a promising reporter gene with the potential for visualizing and assessing CAR-T cells. Emami-Shahri and colleagues 58 used an hNIS/^99m^TcO_4_^-^ reporter gene/probe pair for imaging and monitoring prostate-specific membrane antigen (PSMA)-targeting CAR-T cells. They transduced hNIS into specific PSMA-targeting CAR-T (4P28ζN^+^) cells, which did not reduce their expression of CAR, and did not interfere with their efficient killing properties against the PSMA-overexpressing prostate cancer cell line PC-LN-PSMA *in vitro* as well as against tumor-bearing mice *in vivo*. Serial SPECT/CT imaging for 4P28ζN^+^ or control (4PTrN^+^, non-PSMA-targeting) T cells in tumor-bearing mice was performed over 14 d p.t, showing increased ^99m^TcO4^-^ activity in tumors treated with 4P28ζN^+^ T cells and no significantly increased signal in tumors treated with 4PTrN^+^ T cells. These data not only provided evidence that T cells could migrate to the tumor and infiltrate tumor tissue, but that specific CAR-T cells targeting PSMA have stronger tumor homing ability in comparison to control CAR-T cells (**Figure 5**).

**Figure 6:**
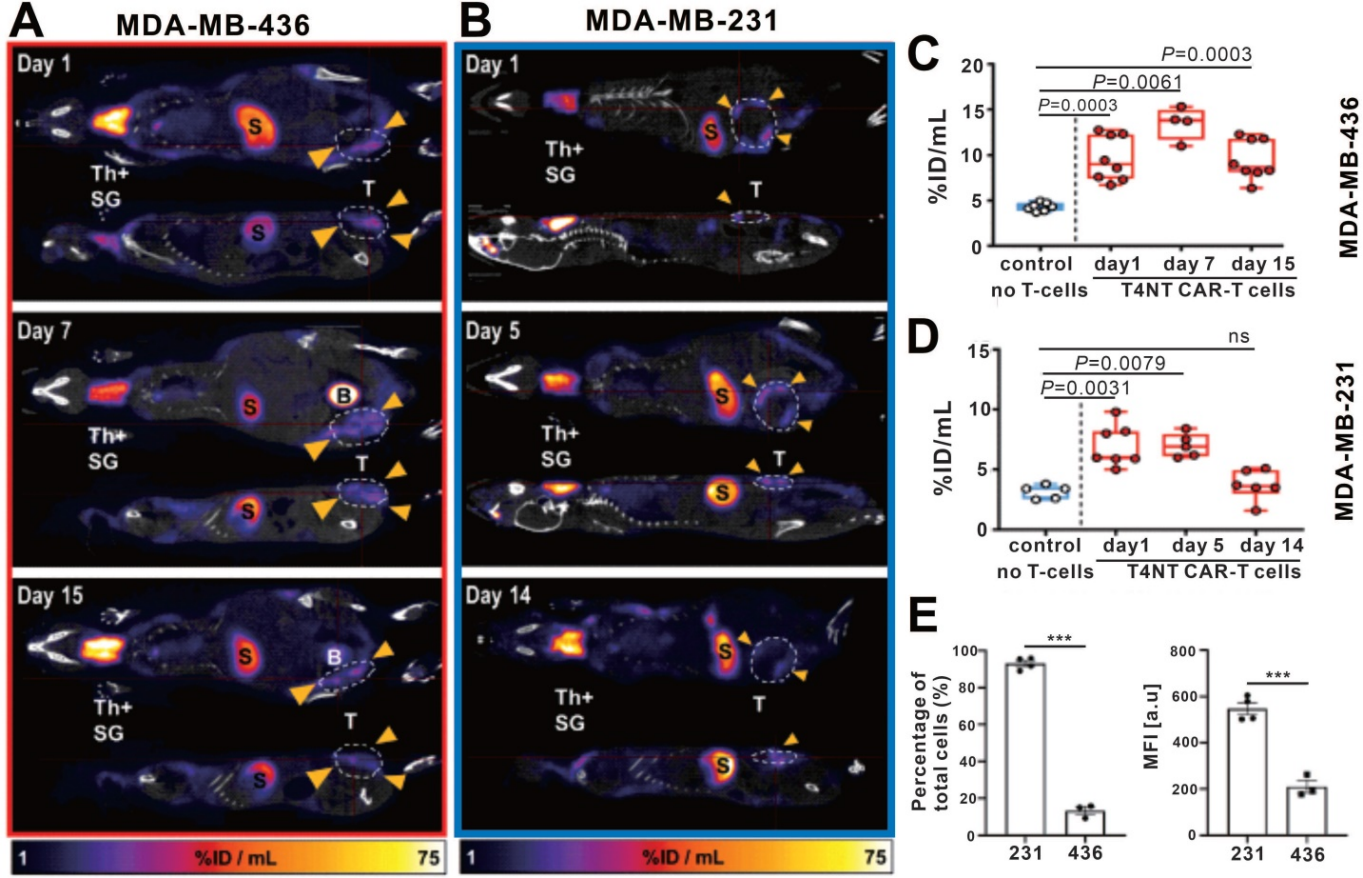
** CAR-T retention in tumor visualized on hNIS reporter imaging. A-B:** ^18^F-BF4^-^ PET/CT imaging of MDA-MB-436 and MDA-MB-231 xenograft-bearing mice. **C-D**: The PET signal intensity of the ROI of the MDA-MB-436 and MDA-MB-231 xenografts. Physiological uptake in the thyroid (Th+), salivary glands (SG), stomach (S), and bladder (S) on PET/CT images was noted in both MDA-MB-436 and MDA-MB-231 xenograft-bearing mice. The CAR-T retention in tumor (circles and arrowheads) differed: CAR-T signal was always observed in MDA-MB-436 xenografts (**A**), and remained at a high level until day 15 p.t (**C**); while the CAR-T signal in MDA-MB-231 xenografts decreased over time (**B** and** D**). **E.** Significantly higher PD-L1 expression in MDA-MB-231 compared with MDA-MB-436 cells was observed. Adapted with permission from 105, copyright 2020 The American Society of Gene and Cell Therapy.

Volpe et al. used the PET probe ^18^F-BF4^-^ to reaffirm the role of the NIS reporter system in trafficking CAR-T cells 105. They constructed T4NT CAR-T cells that express the NIS reporter and can target the pan-ErbB family. The cell uptake assay showed that the CAR-T cells had significantly higher *in vitro* uptake of SPECT radiotracer ^99m^TcO4^-^ and PET radiotracer ^18^F-BF4^-^ in comparison to NIS-free T cells. And after radiation exposure, the cellular viability, IFN-γ secretion ability and tumor killing ability of CAR-T cells were not significantly affected. Notably, the researchers observed that when CAR-T cells exposed to radioactive probes, large doses of ^99m^TcO4^-^ (>51mBq/cell) and ^18^F-BF4^-^ (>14mBq/cell) will induce DNA damage in the early stage (at 2h), but radiation-induced damage was repaired within 24 h. Two orthotopic triple-negative breast cancer (MDA-MB-436 and MDA-MB-231)-bearing NSG mice received 5 × 10^6^ T4NT CAR-T cells by intratumoral injections, and then underwent serial small animal PET/CT imaging (on day 1, 7, and 15 p.t for MDA-MB-436; on day 1, 5, 14 for MDA-MB-231). Surprisingly, CAR-T retention was observed in MDA-MB-436 xenografts up to the day 15 p.t, whereas CAR-T cells in MDA-MB-231 xenografts disappeared over that time (**Figure 6A-D**). The author believes that this difference in CAR-T retention is likely to be intrinsic to the tumor cells, that is, from the influence of immune checkpoints. They further detected the expression of programmed death ligand 1 (PD-L1) in the two tumors, and an inverse correlation between T4NT CAR-T cell retention and the immune checkpoint inhibitor PD-L1 expression was observed (**Figure 6E**). This study achieved PET-based NIS reporter gene imaging with a higher detection sensitivity comparing to the aforementioned SPECT imaging (3,000 cells *versus* 15,000 cells) 58. They concluded that immune checkpoints affect CAR-T cells retention in different tumors, and highlighted the multifactorial challenges facing CAR-T therapy in solid tumors.

### Receptor-based reporter gene

**Figure 7:**
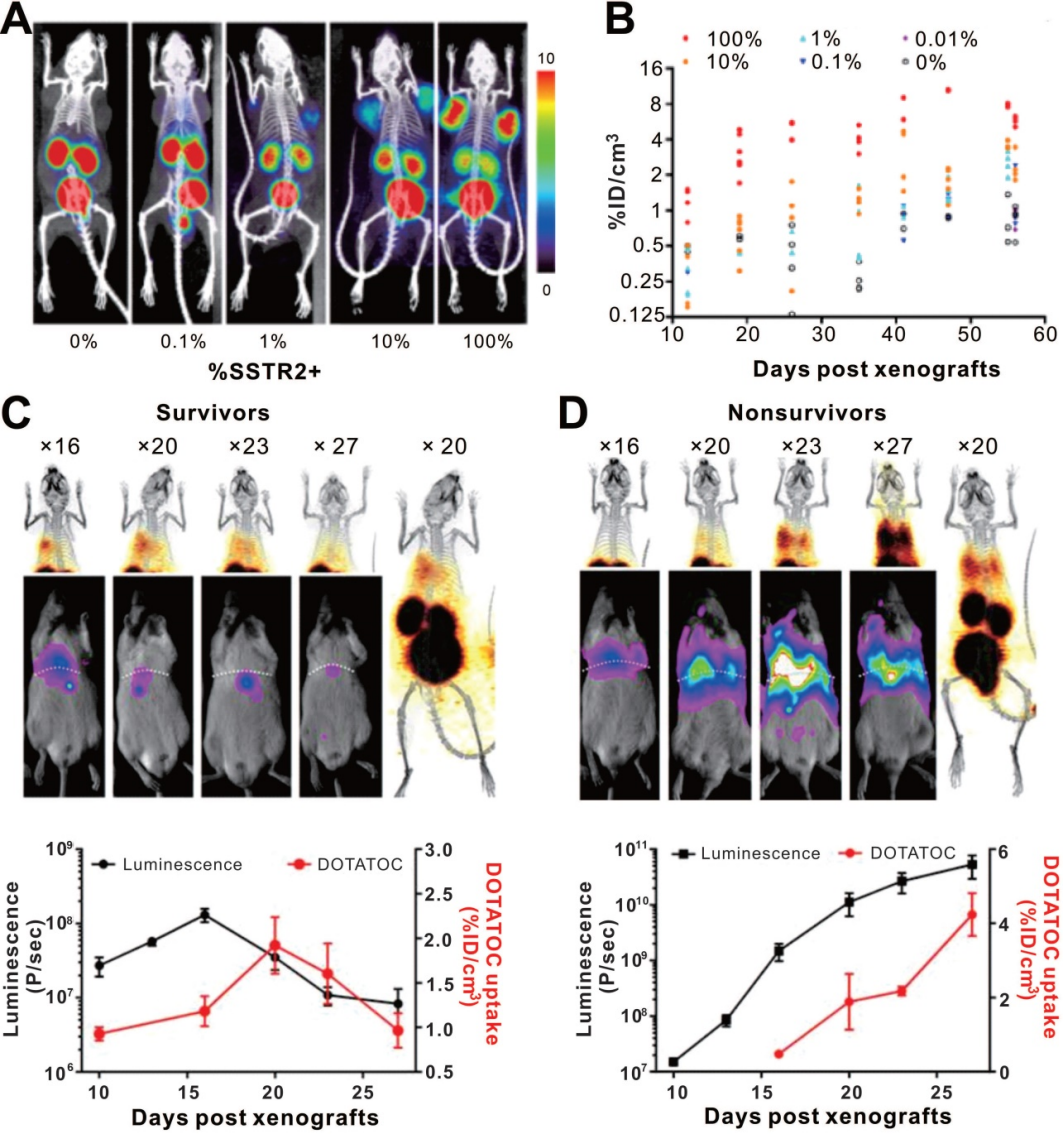
** CAR-T cellular visualization based on SSTR reporter imaging. A-B.** SSTR2+ CAR-T cell detection limit of ^68^Ga-DOTATOC PET/CT. Jurkat T cell clusters mixed with different proportion of CAR-T cells were inoculated into the shoulders of NSG mice, which were imaged with ^68^Ga-DOTATOC PET/CT. As low as a 1% SSTR2^+^ CAR-T cell cluster was visible (A), and the ^68^Ga-DOTATOC uptake increased following cell proportion increase and over time (B). **C-D.** Serial BLI/PET images (upper row) and corresponding time-signal curves (bottom row) of survival (left column) and non-survival (right column) mice with 8505c-FLuc^+^GFP^+^ cells *in situ* lung tumor after SSTR2^+^ ICAM-1-targeting CAR-T treatment (the labels “× 16, 20, 23…” refer to the day 16, 20, 23...post xenograft modeling). The survivors' images exhibited an increase in uptake followed by a decline. Conversely, the BLI and PET signals continually increased in non-survivors. Adapted with permission from 109, copyright 2016 American Society for Clinical Investigation.

Somatostatin receptors (SSTRs) belong to the G protein-coupled receptor family, which are highly conserved in mice and humans with relative low expression in most organs. SSTRs contain five distinct subtypes (termed SSTR1, 2, 3, 4, and 5). SSTR2 exhibits the highest affinity for natural somatostatin and synthetic somatostatin analogs 106, 107. A major advantage of the SSTR2 reporter system is its compatibility with multiple probes such as ^68^Ga labeled 1,4,7,10-tetraazacyclododecane-N^I^, N^II^, N^III^, N^IIII^-tetraacetic acid-d-Phe^1^-Tyr^3^-octreotate (DOTATATE) and 1,4,7,10-tetraazacyclododecane-N^I^, N^II^, N^III^, N^IIII^-tetraacetic acid (D)-Phe^1^-thy^3^-octreotide (DOTATOC) for PET and ^99m^Tc-Hynic-octreotide for SPECT 108. Vedvyas et al. engineered native human T cells to co-express intercellular adhesion molecule-1 (ICAM-1) for tumor targeting and SSTR2 for reporting 109. The authors established a simple method for estimating the density of SSTR2-expressing CAR-T cells infiltrating in solid tumors (**Figure 7**). Interestingly, the authors revealed a biphasic CAR-T cell expansion and contraction pattern in the survivors, almost matching tumor growth and destruction, whereas the non-survivors had unabated T cell expansion because the tumor-killing effect of the CAR-T cells could not overcome more intense tumor growth. Although this study provides a visual tool for quantifying T cell infiltration and assessment of prognosis basing on SSTR2 reporter gene imaging, several shortcomings of this reporter system need to be mentioned: i) SSTR2 receptor is expressed endogenously on various immune cell types including T-cells, B-cells and macrophages, decreasing the reporter imaging specificity and possibly interfering with immune function 107, 110, 111; ii) it is also expressed in the gastrointestinal tract; and iii) the SSTR2 receptor will be internalized upon ligand binding 108, 112.

**Figure 8:**
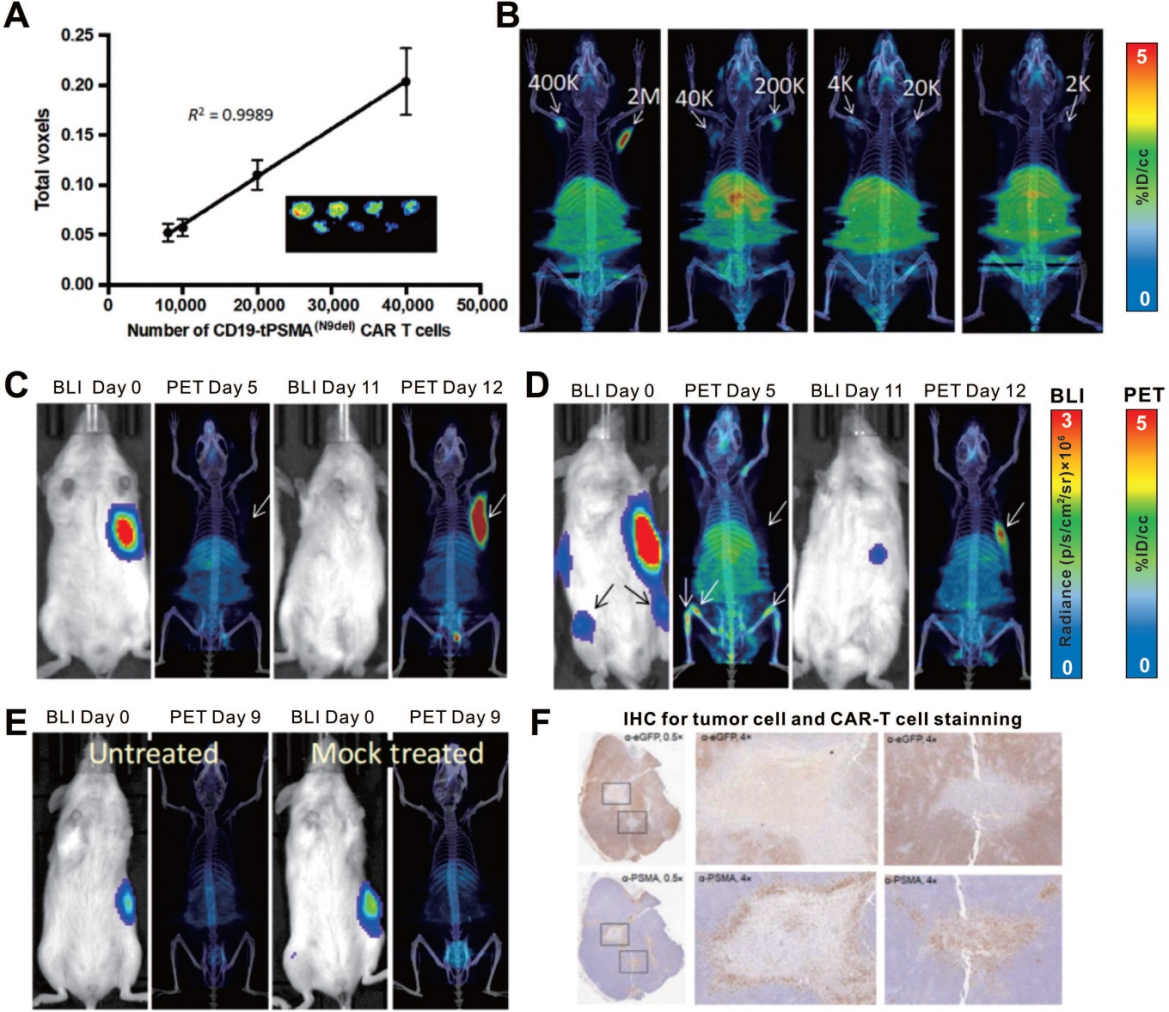
** CAR-T cellular visualization based on PSMA reporter imaging. A-B.** The *in vitro* (A) and *in vivo* (B) detection limit of ^18^F-DCFPyL for CD19-tPSMA CAR-T cells.** C-E.** NSG mice with CD19^+^Nalm6-enhanced green fluorescent protein (eGFP)-fLuc xenograft at the right frank (C, D, and E) and bone metastases (D) were treated with CD19-tPSMA CAR-T cells (C, D), untransduced T cells (E, right panel), and no cells treated (E, left panel), and then underwent BLI for visualization of tumor cells and ^18^F-DCFPyL PET/CT for T cells alternately. The initial BLI confirmed tumor burden, and PET/CT in the early stage showed no observable signal in the xenograft; the PET/CT on the day 12 p.t exhibited CAR-T cells infiltration following by tumor regression (C). There was no significant PET signal in the subcutaneous xenograft whereas activity was detected in the bone metastases on the day 5 p.t; repeated BLI showed only subcutaneous remnant, with elevated PET signal accumulation (D). No PET signal was detected in the tumors, either in mice treated with mock T cells or in untreated mice (E). **F**. IHC confirmed CD19-tPSMA CAR-T cells infiltrating the xenograft. Adapted with permission from 59, copyright 2018 American Association for the Advancement of Science.

PSMA is a non-immunogenic human protein whose tissue expression is normally restricted to a few organs such as the prostate, kidneys, and brain 113, 114; it is a widely used target for various imaging modalities 115, 116. However, internalization of wild-type PSMA upon ligand binding prevents its clinical translation as a reporter gene. An N-terminally modified PSMA variant, tPSMA^(N9del)^, was designed by Il Minn and colleagues to prevent PSMA internalization and increase surface expression 117, which enhanced the binding and overall imaging sensitivity of the PET probe 2-(3-{1-carboxy-5-[(6-^18^F-fluoro-pyridine-3-carbonyl)-amino]-pentyl} F-ureido)-pentanedioic acid (^18^F-DCFPyL). Il Minn et al. also recently applied the tPSMA^(N9del)^/^18^F-DCFPyL pair for CD19-targeting CAR-T monitoring 59. ^18^F-DCFPyL exhibited effective binding to PSMA because of the high number of target sites per cell (approximately 1 × 10^6^), enabling the visualization of as few as 2000 cells *in vitro* or *in vivo* (**Figure 8A-B**). This detection limit provides the potential to track the kinetics of a small number of CAR-T cells. The authors established subcutaneous xenografts and osseous metastases in NSG mice by inoculating them with CD19^+^ Nalm6 cells. They performed serial ^18^F-DCFPyL PET/CT imaging to visualize CD19 CAR-T infiltration in local and metastatic tumors (**Figure 8C-F**). They also showed that the number of CAR-T cells in the peripheral blood and bone marrow did not correlate with the number of cells infiltrating the tumors, indicating that both peripheral blood tests and bone marrow biopsies may not truly reflect the extent of CAR-T tumor infiltration.

In addition to the aforementioned SSTR2, hNIS, HSV1-tk, and PSMA, another human reporter gene, human norepinephrine transporter (hNET) has been used for T cell visualization 118. The advantage of hNET as a reporter is that its corresponding radiolabeled probe, metaiodobenzylguanidine (MIBG), is currently used in the clinic, and can be radiolabeled with ^123^I or ^131^I for SPECT and γ-camera imaging and with^124^I for PET imaging 119-121. A comparative study 62 to investigate the sensitivity of four different reporter gene systems (HSV1-tk, hNET, hNIS, and human deoxycytidine kinase double mutant [hdCKDM]) in detecting reporter transduced T cells showed that the hNET reporter system is the most sensitive and capable of detecting approximately 3.5-4.0 × 10^4^ T cells at the site of T cell injection in mice. This makes it a potential candidate for CAR-T visualization; whether it has clinical translation potential will require further experimental data.

### Antibody-based reporter genes

**Figure 9:**
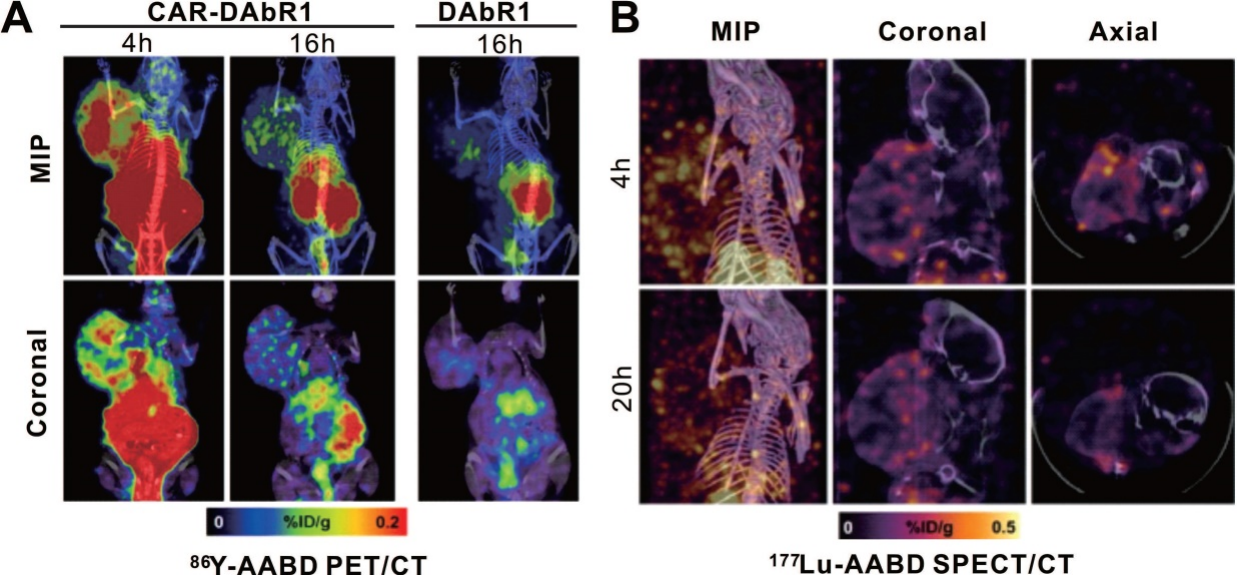
** CAR-T cellular visualization based on DAbR1 reporter imaging. A.** Adoptively transferred T cells tracking by ^86^Y-AABD PET/CT. Maximum intensity projection (MIP) images (top row) and selected coronal (bottom row) images showed significant activity accumulation in tumors treated with CAR-DAbR1 T cells whereas no uptake observed in tumors treated with DAbR1 T cells. **B.** Transferred T cells tracking by ^177^Lu-AABD SPECT/CT. MIP, coronal, and axial SPECT/CT images of tumor-bearing mice demonstrated the CAR-DAbR1 T cells localized in the tumors. Adapted with permission from 125, copyright 2018 Society of Nuclear Medicine and Molecular Imaging.

Krebs and colleagues used a murine-derived single-chain antibody variable fragment (scFv) fragment to construct 1,4,7,10-tetraazacyclododecane-1,4,7,10-tetraacetic acid (DOTA) antibody reporter 1 (DAbR1) as a reporter, based on previous work 122-124. It can bind irreversibly to lanthanoid-(S)-2-(4-acrylamidobenzyl)-DOTA (AABD). Krebs et al. 125 used DAbR1/AABD as a reporter/probe pair for CD19-targeting CAR-T cells tracking *in vivo*. The authors labeled AABD with ^86^Y and ^177^Lu for PET/CT and SPECT/CT imaging, respectively. They first verified the feasibility of ^86^Y-AABD PET/CT to visualize DAbR1-expressing CAR-T cells, then showed that whether the T cells were infused subcutaneously or intratumorally into subcutaneous U373 glioma xenografts, obvious PET signal emitted by CAR-T cells was noted. Mice with Nalm6 subcutaneous xenografts were treated with DAbR1-transduced, CAR-DAbR1-transduced, and non-transduced T cells, respectively. Both PET and SPECT showed that CAR-DAbR1 T cells aggregated in the high-CD19-expression tumors with favorable biodistribution and high image contrast, indicating the DAbR1 reporter imaging with PET/SPECT can localize and track CAR-T cells (**Figure 9**). The authors assessed the absorbed dose to T cells as approximately 20 cGy following a dose of 3.7 MBq of ^86^Y-AABD administration, significantly below the previously reported tolerated dose (830 cGy) 126, which shows the safety of long-term monitoring of CAR-T cells using these radiotracers. In addition, considering the potentially lethal side effects of adoptively transferred CAR-T cells, the authors also explored DAbR1 as a suicide gene. They stated that the CAR-T cell cluster might receive an absorbed dose of 1.1 Gy/MBq following ^177^Lu-AABD injection, which is a radiotoxic dose, indicating that T cells would be damaged or killed by the radiation.

The ideal reporter system for imaging should meet several conditions: (1) reporter gene expression should be low in normal tissues to avoid background interference and to obtain a good signal-to-noise ratio; (2) reporter-encoded products should have high specificity and affinity for the probe to improve imaging sensitivity and specificity; (3) the products should not be immunogenic, to avoid the risk of transduced T cells being attacked and cleared by the immune system; (4) a reporter should not affect the viability and function of engineered cells; and (5) it should have the ability to be imaged using a readily-available radionuclide with a clinical imaging modality. The reporter systems currently used for CAR-T therapy monitoring have one or more limitations, including the potential immunogenicity of HSV-1 tk, eDFHR, and DAbR1; and inherent expression in multiple normal organs resulting in background interference such as the case with SSTR2, hNIS, and PSMA. In addition, the reporter gene strategy has several common disadvantages, including complexity and difficulty in design and operation, promoter silencing, and potential mutation caused by DNA modification 127, 128. In summary, reporter gene imaging can non-invasively visualize and monitor CAR-T cells *in vivo* with a relatively unrestricted time window, which is undoubtedly the most promising technique for clinical translation. Several key points require the most attention, that is, the safety, imaging sensitivity and specificity, the selection of an appropriate reporter system, and a protocol designed to address actual clinical issues.

## Endogenous cell imaging methods

Another imaging strategy, endogenous T cell imaging, can map cell distribution *in vivo* and reflect cellular activation and functional status. To date, it is the most mature technology implemented in clinical research 35, 46. Although there are few CAR-T-related studies in this field so far, it provides an alternative approach for *in vivo* imaging of CAR-T cells, which can be used as a supplement to direct labeling and reporter gene-based cellular imaging strategies.

### Cell surface marker-based imaging

Cell surface markers are meaningful targets for *in vivo* imaging of specific T cell populations. These targets include general markers such as CD4, CD8, CD3, and immune checkpoints such as programmed death 1 (PD-1), PD-L1, and cytotoxic T-lymphocyte-associated protein 4 (CTLA-4) 129. By constructing radionuclide probes that can specifically target these markers, PET/SPECT imaging can be used to trace target cell populations, evaluate the expression level of their markers, and reflect immune status. The construction of corresponding probes is based on the radiolabeling of antibodies or antibody-derived constructs and engineering bivalent antibody fragments, which build upon scFv derived from intact antibody, such as cys-diabodies (dimer of scFv) and minibodies (dimer of scFv-C_H_3) 46, 130, 131.

Larimer et al. 132 used a murine ^89^Zr-labeled anti-CD3 antibody (^89^Zr-DFO-CD3) to quantify T cell infiltration in colon cancer during anti-CTLA-4 treatment. High levels of infiltration were found to precede tumor regression. Rashidian et al. 133 developed an ^89^Zr-labeled PEGylated single-domain antibody fragment specific for CD8, which detected CD8^+^ tumor-infiltrating lymphocytes in melanoma and pancreatic cancers, and distinguished therapy-responsive *versus* nonresponsive tumors tissue through longitudinal immunoPET. In preclinical models, both ^89^Zr-labeled anti-CD4 and anti-CD8 cys-diabodies have been used to target and visualize respective T cell populations 134. There is concrete evidence that CD4^+^ and CD8^+^ lymphocytes increasingly infiltrating the tumor microenvironment connote a response to immunotherapy and a favorable prognosis 135.

Tumor cells can upregulate the expression of PD-L1, which interacts with PD-1 on T cells 136. The PD-1/PD-L1 interaction plays a major role in the inhibition of T cell activity 135, 136. Preliminary clinical studies 68, 137 showed that ^89^Zr-Df-nivolumab PET was a safe and feasible method to map the localization of PD-1-expressing T cells and to assess their PD-1 expression *in vivo*; a correlation between ^89^Zr-Df-nivolumab uptake intensity and the number of PD-1-expressing lymphocytes in biopsied tumor was observed. Higashikawa et al. developed a CTLA-4-targeting PET probe (^64^Cu-DOTA-anti-CTLA-4 mAb) to examine the expression of CLTA-4 in CT26 tumor-bearing mice 138. They found that tumor-infiltrating T cells were responsible for the high CTLA-4 expression.

A recent investigation from Xiao et al. identified Inducible T-cell COStimulator (ICOS or CD278) as a costimulatory molecule upregulated during T cell activation 47. They designed an ICOS-targeting tool (^89^Zr-DFO-ICOS mAb PET) for visualizing the activation of T cells and prediction of therapeutic response in the Lewis lung cancer model. Based on this work, ^89^Zr-DFO-ICOS mAb PET/CT has recently been used to visualize CAR-T cell activation. Significantly higher PET signal was detected in the bone marrow of mice with systemic B-cell lymphoma mice treated with CD19 CAR-T cells, which reflected their distribution and activation 139.

### Metabolism-based T cell imaging

When activated, T cells are metabolically programmed to switching on additional metabolic processes and upregulate substrate inflow, which allows them to develop full effector phenotypes, and survive and function in peripheral tissues 46. Targeting these metabolic pathways can help to distinguish between activated and non-activated T cells, as well as to assess ensuing immune response at early time point.

Several tracers have been developed as substrates for enzymes, such as deoxycytidine kinase (dCK) and deoxyguanosine kinase (dGK), which are rate-limiting enzymes in the deoxyribonucleoside salvage pathway 46. ^18^F-CFA (^18^F-clofarabine) is a nucleotide purine analogue metabolized *via* dCK, which has preferential *in vivo* distribution in hematopoietic bone marrow and secondary lymphoid organs 140. ^18^F-AraG (^18^F-Arabinosyl guanine), another PET probe that targets specific metabolic pathways of T cells, accumulates in activated T cells, mainly *via* the dGK pathway 141. ^18^F-AraG PET imaging of a murine acute graft-*versus*-host-disease (aGVHD) model has been shown to visualize the activation of donor T cells in secondary lymphoid organs prior to the appearance of aGVHD symptoms 142. Other PET tracers that target metabolic pathways, such as ^18^F labeled fluorodeoxyglucose (FDG) and fluorothymidine (FLT), can also potentially monitor multiple cell types involved in innate and adaptive immunity and measure cell function 143.

In summary, endogenous cell imaging has shown the feasibility of direct assessment of the presence, localization, numbers, activation and functional status of specific cell populations such as CTLs *in vivo* during immunotherapy 35, 46, 141, which can provide key information about the role of CAR-T cells in cellular immunotherapy, and promote the understanding and improvement of CAR-T immunotherapy. Nevertheless, the chief drawback to the application of endogenous cell imaging methods for CAR-T visualization is the nonspecific uptake of probe by the endogenous T cells, which cannot be distinguished from infused CAR-T cells.

## Current challenges and strategy choice

The preclinical studies 48, 54 and clinical trials 64, 77 discussed above have already answered some of the questions about CAR-T monitoring raised in the Introduction. Both direct imaging and indirect imaging can provide spatial information on the transferred cells. This is beneficial not only to observe the trafficking, homing, expansion, contraction, and retention of cells through PET/SPECT signal detection and quantification, but also to determine whether there is nonspecific uptake in normal tissues, which is crucial for assessing the off-tumor/on-target toxicity. PET and SPECT images, especially fusion images integrated with CT or MRI, can provide tomographic and anatomic information. This is of great help in mapping the accurate spatial distribution of CAR-T cells in multiple dimensions, and in assessing whether CAR-T cells are infiltrating tumors. In addition, the activation and functional status of T cells can be monitored through metabolic pathway-/cell marker-targeting imaging 137, 139. Notably, judging whether the expansion or contraction of cells matches the change in tumor burden can indirectly reflect ineffective proliferation (T cells continue to proliferate, but the tumor is not controlled) or effective killing (the tumor shrinks after T cell expansion) 109.

The choice of imaging strategy depends on several key aspects, including imaging technology, modality, and radionuclide. Compared with direct labeling and endogenous T cell imaging, reporter-based imaging is a more promising technique for visualizing and monitoring of CAR-T cells due to the advantages mentioned above. Nevertheless, two issues require scrutiny: immunogenicity and inherent expression of reporter genes. Focusing on identification of new human reporter genes may address the former issue. Choosing an appropriate reporter gene system for a specific clinical setting can help to weaken the influence caused by endogenous expression of reporter gene. For example, although hNIS is highly expressed in the thyroid, salivary glands and stomach 89-91, we can still apply hNIS-based reporter imaging for trafficking the CAR-T cells in other organs without hNIS expression, which provides a low background so that CAR-T cells can be detected easily.

The choice of imaging modalities (PET or SPECT) is also of great significance for the choice of imaging instrument and corresponding nuclides. The spatial resolution of PET (6-10 mm) is slightly better than that of SPECT (7-15 mm), and the sensitivity is an order of magnitude higher than that of SPECT (10^-11^-^-12^ *versus* 10^-10^-^-11^ mol/L probe) 29. In terms of radionuclides, ^18^F and ^68^Ga would be good choices due to their accessibility. They have relative shorter half-lives, which are practical for human PET imaging. ^89^Zr and ^111^In have longer half-lives, which is beneficial for direct labeling methods to broaden their imaging time window 48, 52. However, reporter gene imaging can be performed repeatedly, and patients will not need frequent repeat imaging with a very short interval. Therefore, long half-life raidonuclides may increase the radiation dose, and not add value for trafficking T cells.

The choice of precursor (or nuclide carrier) also has a major impact on imaging. Although nanomaterials are highly modifiable and have diversified functions, their potential toxicity is always a thorny issue for researchers. Therefore, human imaging is mainly based on small molecules. Since previous data have confirmed their safety and feasibility, the small molecule probes that have been used in clinical practice (such as ^68^Ga-DOTATOC and ^18^F-DCFPyL) will be more widely used. If researchers design a new imaging probe, some important characteristics should be taken into consideration, such as multiple radionuclide compatibility, fast clearance *in vivo*, low cytotoxicity, and a high target-to-background ratio.

## Conclusions and Perspective

A variety of strategies have been developed to enhance the therapeutic effect of CAR-T in solid tumors, such as potentiating the tumor killing 144, 145, improving the migration capacity 146, 147, infiltration ability 148, 149, and safety 150, as well as combining with radiotherapy 151, 152 or immune checkpoint blocking treatment 153, 154. However, whichever strategies are employed, CAR-T cell monitoring is required for in-depth understanding of the therapeutic mechanism and to provide the basis for optimization. Noninvasive imaging, especially the reporter gene imaging approach, provides a tool for continuous and dynamic assessment of the distribution, migration, and function of human CAR-T cells. This field is gradually maturing and exhibits strong clinical translation potential. For certain clinical scenarios, the development of novel reporter gene systems or the rational selection of existing reporter/probe pairs is a key step to addressing the issues that arise during the continuous exploration and optimization of CAR-T therapy in solid tumors.

## Figures and Tables

**Figure 4:**
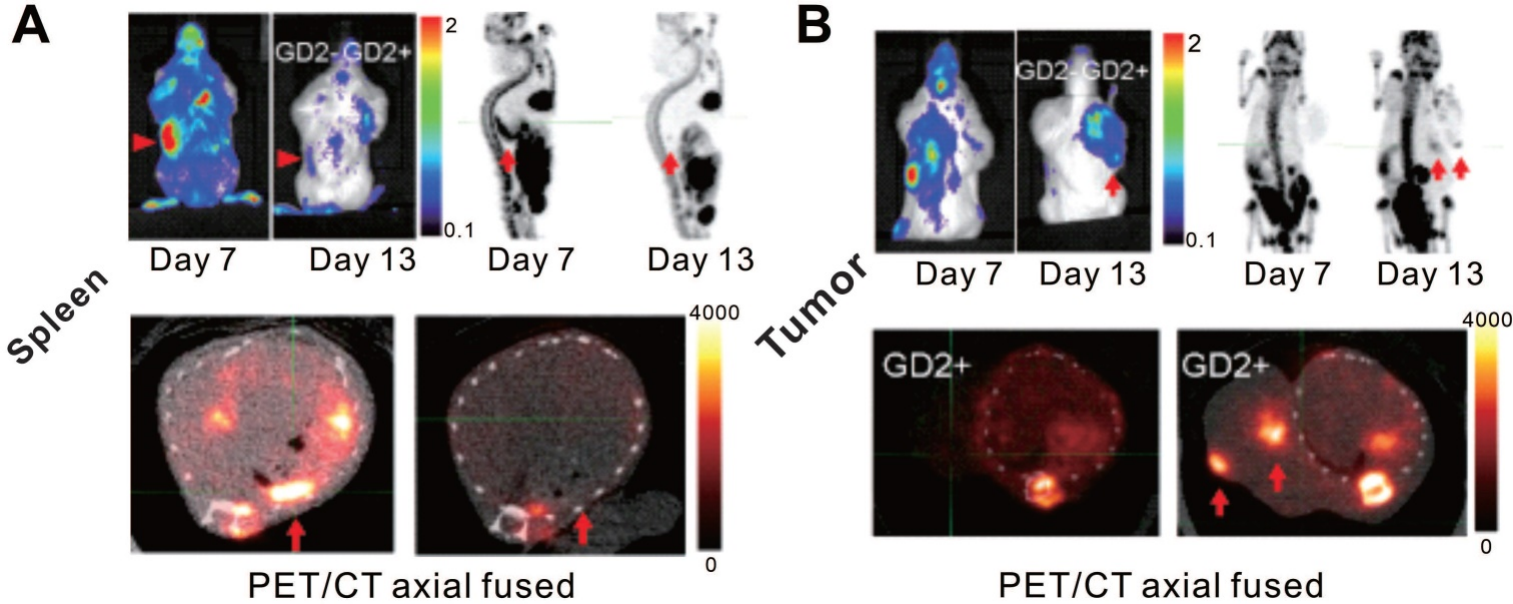
** CAR-T cellular visualization based on eDHFR reporter imaging.** After receiving eDHFR-expressing anti-GD2 CAR-T cells, mice bearing both GD2^-^ HCT116 (left shoulder) and GD2^+^ 143b tumor (right shoulder) were imaged with BLI and ^18^F-TMP PET for CAR-T cellular visualization. The images showed elevated BLI and PET signal in the spleen on day 7 post T cells infusion (p.t), which decreased on day 13 p.t (**A**, red arrows). Neither GD2^-^ nor GD2^+^ tumor displayed BLI or PET signal on day 7 p.t, whereas increased BLI and PET signal was detected only in GD2^+^ tumor (**B**, red arrows) on day 13 p.t. Adapted with permission from 84, copyright 2019 The American Society of Gene and Cell Therapy.

**Table 1:** CAR-T imaging in animal models based on reporter gene strategy

Reporter	Reporter type	Probe & Dose	CAR-targeting	CAR-T infused dose	Transduction method	Tumor cell line	Tumor modeling (inoculated tumor cells number, modeling route)	Imaging Modality	Detection limit(cell number)	CAR-T detection (time)	Ref.
eDHFR	Enzyme-based	^18^F-TMP 3.7 MBq	GD2	1×10^6^	Lentivirus vector	143b (human osteosarcoma)	10×10^6^ cells Subcutaneous xenograft	PET/CT	11,000*	Up to 13 d p.t**	84
hNIS	Symporter-based	^99m^TcO_4_- 20MBq	PSMA	1×10^6^	Lentivirus vector	PC-LN3-PSM (prostate cancer)	2.5×10^5^ cells Subcutaneous xenograft	SPECT/CT	15,000	Up to 14 d p.t	58
hNIS	Symporter-based	^18^F-BF_4_- 5 MBq	Pan-ErbB	5×10^6^	Lentivirus vector	MDA-MB-436 & MDA-MB-231 (triple negative breast cancer)	1×10^6^ cells Orthotopic mammary xenograft	PET/CT	3,000	Up to 14 and 15 d p.t***	105
SSTR-2	Receptor-based	^68^Ga-DOTATOC#	ICAM-1	2-3×10^6^	Lentivirus vector	8505C (thyroid cancer)	1×10^6^ cells Tail intravenous Primary lung tumor & hepatic/distant metastases	PET/CT	50,000	Up to 27 d p.m.##	109
tPSMA^(N9del)^	Receptor-based	^18^F-DCFPyL 14.8 MBq	CD19	2×10^6^	Lentivirus vector	Nalm6 (acute lymphoblastic leukemia)	1×10^6^ cells Subcutaneous xenograft	PET/CT	2,000	Up to 12 d p.t	59
DAbR1	Antibody-based	^86^Y-AABD 3.7 MBq	CD-19	3×10^6^	Retrovirus	Nalm6 (acute lymphoblastic leukemia)	5×10^6^ cells Subcutaneous xenograft	PET/CT	Not available	4h, 16 h p.t	125
		^177^Lu-AABD 37 MBq						SPECT/CT		4h, 20 h p.t	

*The detection limit is 11,000 cells/mm^3^; **p.t: post T cells infusion; ***: 14 d p.t for MDA-MB-436 xenografts, and 15 d p.t for MDA-MB-231 xenografts; #: the dose of ^68^Ga-DOTATOC is not available; ##p.m.: post modeling.

## Abbreviations

ACT: adoptive cell therapy
CAR: chimeric antigen receptor
IFN: interfron
IL-2: interleukin 2
CXCR-2: C-X-C chemokine receptor type 2
CT: computed tomography
MRI: magnetic resonance imaging
OI: optical imaging
BLI: bioluminescence imaging
FLI: fluorescence imaging
PET: positron emission tomography
SPECT: single photon emission computed tomography
SCID: severe combined immunodeficiency
IL13Rα2: interleukin-13 receptor α2
PSCA: prostate stem cell antigen
NSG: nonobese diabetic severe combined immunodeficient
GNP: gold nanoparticle
DOTA: 1,4,7,10-tetraazacyclododecane-1,4,7,10-tetraacetic acid
PEG: polyethyleneglycol
DNA: Deoxyribonucleic acid
HSV-1 tk: herpes simplex virus 1 thymidine kinase
FIAU: 2-fluoro-2-deoxy-1-β-D-arabinofuranosyl-5-iodouracil
FHBG: 9-(4-fluoro-3-hydroxymethylbutyl)guanine
FEAU: 2-deoxy-2-fluoro-5-ethyl-β-D-arabinofuranosyl-uracil
FHPG: 9-(3-fluoro-1-hydroxy-2-propoxymethyl) guanine
CTL: cytotoxic T lymphocyte
TCR: T-cell receptor
NFAT: nuclear factor of activated T cells
IL-13: interleukin 13
SUVmean: mean standard uptake value
eDHFR: *E. coli* dihydrofolate reductase
TMP: fluoropropyl-trimethoprim
p.t: post T cells infusion
hNIS: human sodium-iodine symporter
PSMA: prostate-specific membrane antigen
PD-L1: programmed cell death protein ligand 1
SSTR: somatostatin receptor
DOTATATE: 1,4,7,10-tetraazacyclododecane-N^I^,N^II^,N^III^,N^IIII^-tetraacetic acid-d-Phe1-Tyr3-octreotate
DOTATOC: 1,4,7,10-tetraazacyclododecane-N^I^,N^II^,N^III^,N^IIII^-tetraacetic acid (D)-Phe1-thy3-octreotide
ICAM-1: intercellular adhesion molecule-1
^18^F-DCFPyL: 2-(3-{1-carboxy-5-[(6-^18^F-fluoro-pyridine-3-carbonyl)-amino]-pentyl}-ureido)-pentanedioic acid
eGFP: enhanced green fluorescent protein
hNET: human norepinephrine transporter
MIBG: metaiodobenzylguanidine
hdCKDM: human deoxycytidine kinase double mutant
DAbR1: DOTA antibody reporter 1
AABD: lanthanoid-(S)-2-(4-acrylamidobenzyl)-DOTA
MIP: maximum intensity projection PD-1 programmed cell death protein 1
CTLA-4: cytotoxic T-lymphocyte-associated protein 4
scFv: single chain variable fragment
ICOS: inducible T-cell COStimulator
dCK: deoxycytidine kinase
dGK: deoxyguanosine kinase
aGVHD: acute graft-versus-host-disease
FDG: fluorodeoxyglucose
FLT: fluorothymidine
